# Anatomical Bill*Since this article was written we perceive that the bill referred to has passed the Assembly, and been ordered to a third reading in the Senate. We shall endeavor to present its details in our next.

**Published:** 1853-04

**Authors:** 


*Anatomical Bill.** — A bill is now before the legislature of this state, legalizing dissections, and making provisions, with proper restrictions, for supplying medical schools with anatomical material. We observe in one of the reports of the proceedings of the Assembly, that a Mr. Kennedy, when this bill came up, moved "that all doctors who die may be delivered to any other doctor applying for the same within twenty-four hours after, unless requesting burial before death." Now we have no doubt, in Mr. Kennedy's opinion, this motion was wonderfully witty. It is to be presumed he imagined the assembly, and his worthy constituents would be electrified by so admirable a jest. He is evidently a man of no little *smartness* in his own estimation; and one can fancy the self-complacency with which this funny legislator rose and threw off so brilliant a scintillation. We have no disposition to disturb his good opinion of himself as a humorist. Indeed, we are inclined to congratulate him that so nice an opportunity presented itself for the display of his facetious ability. He had an excellent chance to give the doctors a slap, and it would be altogether too much to expect him to resist the temptation. Besides, it is more than probable that in all the graver matters of legislation he is but poorly qualified to cut a conspicuous figure. It would be ungenerous, therefore, to complain of his availing himself of the only field suited to his capacity.

In perpetrating the *jeau d' esprit*, however, Mr. Kennedy gives utterance to an idea in which he is not alone. The idea involved in his elegant sally is, that legalized facilities for dissection are petitioned for by the doctors with reference to their own exclusive advantage, not for the benefit of the public.

---

* Since this article was written we perceive that the bill referred to has passed the Assembly, and been ordered to a third reading in the Senate. We shall endeavor to present its details in our next.

Mr. K. is one of that very intelligent and liberal-minded class who suppose that doctors dissect dead bodies for the love of the thing. They regard a medical man as a species of *ghoul*, and think that in asking the legislature to make provision for the knowledge of practical anatomy, the object is purely that they may enjoy larger opportunities for the gratification of a singular taste for an occupation which other persons view with repugnance, if not with horror. Such a sentiment, we regret to say, is not altogether confined to the facetious Mr. Kennedy.

We venture to *guess* that Mr. K. is one of those who would be sufficiently requiring of his medical attendant in the event of an accident befalling himself which called for accurate anatomical knowledge. We opine that if he got a crooked leg after a bad fracture, the unfortunate doctor employed in the case would think himself well off if he escaped only the worse by a jest or two at his expense. We doubt if Mr. K. would rank himself among the advocates of a repeal of the law rendering a surgeon liable for damages for mal-practice.

We would respectfully submit an argument on the inconsistency of demanding, on the one hand, knowledge and skill which can alone be acquired by dissections, and, on the other hand, imposing the necessity of prosecuting the latter under fearful penalties — but for our professional readers this would be out of place because the matter is to them so palpable, and we cannot flatter ourselves that any remarks we might pen on this subject would affect the judgment of so profound a legislator as the author of the caustic witticism which has occasioned what we have already written.

We cannot refrain from expressing surprise that with Mr. Kennedy's notions of the propensities of doctors, he did not feel some apprehensions lest in their utter inability to cope with his satirical genius, they might be induced to take post-mortem revenge. He could hardly be blind to the danger of coming at last under their scalpels. This shows that he is a man of mettle, as well as wit and wisdom. *Au revoir* Mr. K.

